# Scaling SARS-CoV-2 wastewater concentrations to population estimates of infection

**DOI:** 10.1038/s41598-022-07523-7

**Published:** 2022-03-03

**Authors:** Edward H. Kaplan, Alessandro Zulli, Marcela Sanchez, Jordan Peccia

**Affiliations:** 1 Yale School of Management, Yale University, New Haven, CT 06520 USA; 2 Yale School of Public Health, Yale University, New Haven, CT 06520 USA; 3 Department of Chemical and Environmental Engineering, School of Engineering and Applied Science, Yale University, New Haven, CT 06520 USA

**Keywords:** Environmental sciences, Infectious diseases

## Abstract

Monitoring the progression of SARS-CoV-2 outbreaks requires accurate estimation of the unobservable fraction of the population infected over time in addition to the observed numbers of COVID-19 cases, as the latter present a distorted view of the pandemic due to changes in test frequency and coverage over time. The objective of this report is to describe and illustrate an approach that produces representative estimates of the unobservable cumulative incidence of infection by scaling the daily concentrations of SARS-CoV-2 RNA in wastewater from the consistent population contribution of fecal material to the sewage collection system.

## Introduction

Estimating the unobservable fraction of individuals infected in coronavirus outbreaks is of first-order importance in monitoring epidemic progress and evaluating interventions meant to slow transmission. Given the lack of repeated representative COVID-19 testing over time, researchers have attempted to infer SARS-CoV-2 incidence from observable lagging indicators of infection including clinically diagnosed cases, hospitalizations, and deaths^1–5^. Such indicators present a distorted view of the pandemic due to temporal changes in the rates and coverage of COVID-19 testing, changes in hospital admission and treatment policies, and undercounting of deaths from COVID-19.

Recognizing these difficulties, we initiated daily sampling at a wastewater treatment plant (WWTP) serving a mid-sized US municipality with the objective of obtaining a representative estimate of the unobservable incidence of SARS-CoV-2 infections over time. We previously reported daily SARS-CoV-2 RNA concentrations in this community’s wastewater during the March 2020 epidemic wave, and showed that RNA concentrations followed an epidemic curve while providing an earlier epidemic signal than observed cases or hospitalizations^6^. Via a mathematical epidemic model, we estimated the associated reproductive number *R*_*0*_ and cumulative incidence of infection in this same community^7^.

Building upon this previous work, the objective of this report is to develop and illustrate a simple model that directly scales measured RNA concentrations in sewage sludge to the unobservable fraction of the population infected with SARS-CoV-2 over time. The incidence of infection is determined from the start of the pandemic in Connecticut, USA (March 19, 2020) through May 22, 2021, and the results are compared to three independently developed estimates based on observable cases, hospitalizations, and deaths^3–5^.

## Results

**Figure 1:**
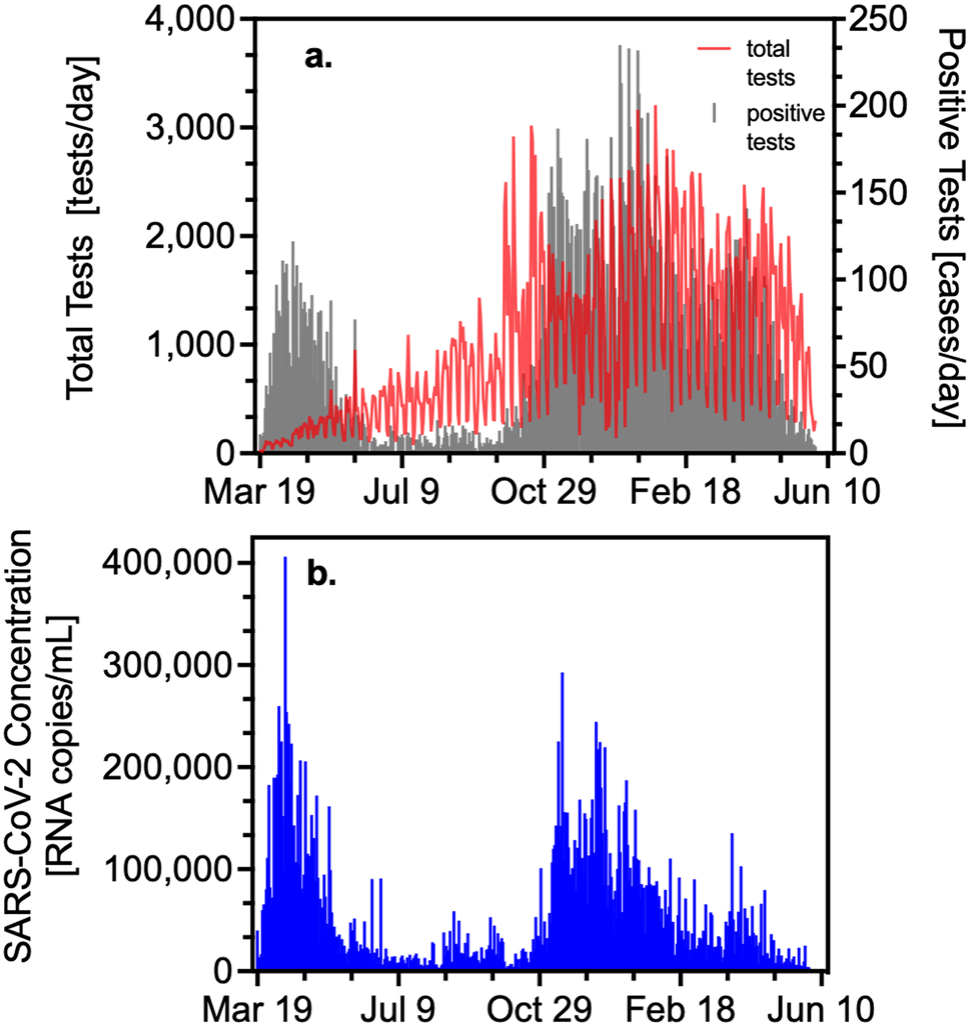
(**a**) Total and positive COVID-19 tests over time. (**b**) SARS-CoV-2 RNA wastewater concentration (copies/mL sludge).

Figure 1a reports the total and positive daily number of COVID-19 tests conducted on the residents of the four towns served by the WWTP. These data demonstrate the large increase in testing as the pandemic progressed, while the positive test results illustrate the two major COVID-19 waves experienced in the area with the second wave yielding higher daily case rates over a longer duration compared to the first. Figure 1b plots SARS-CoV-2 RNA concentration measured in sewage sludge over this same time period. This figure also shows two waves of infection. Unlike the number of positive tests, note that for the wastewater data the first wave, though shorter in duration, peaks at concentrations similar to the second wave. This demonstrates that the early lack of testing impacted the accuracy of reported COVID-19 case information.

**Figure 2:**
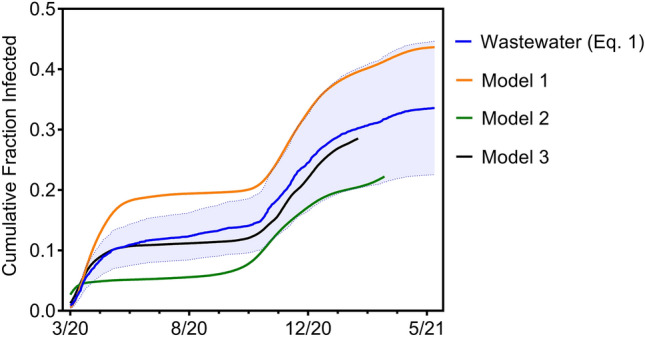
Estimated cumulative incidence of infection with 95% CIs in the population served by the WWTP based on the SARS-CoV-2 RNA concentrations shown in Fig. 1b using Eq. (1). Also shown are cumulative incidence estimates for New Haven County produced by three different statistical models. While all four models show similar trajectories over time, the estimates from Eq. (1) are the middle of the range exhibited by the other models.

Applying Eq. (1) (see “Methods”) to the data contained in Fig. 1b yields the cumulative fraction infected in the population over time (Fig. 2). We estimate that 33.6% (95% CI [24.3%, 42.9%]) of the population was infected by May 22, 2021. By contrast, only 24,296 diagnosed COVID-19 cases were reported in our study population by May 22, 2001, which amounts to 12% of the population. This illustrates the difference between estimating the unobserved incidence of infection and counting the observed number of diagnosed cases of COVID-19. Figure 2 also shows point estimates for the cumulative fraction of the population infected in New Haven County (which subsumes the treatment plant population) produced by three independently developed and computationally intensive statistical models using completely different methods and data sources including COVID-19 cases and deaths (Model 1)^3^; cases, deaths, hospitalizations, and close-contact measures deduced from cell phone geolocation data (Model 2)^4^; and deaths alone (Model 3)^5^. These models demonstrate strikingly similar shapes to and bracket the results of the wastewater-based estimates. Model 1^3^ hugs the wastewater model’s upper 95% confidence limit, Model 2^4^ hugs the wastewater model’s lower 95% confidence limit, and Model^5^ falls just beneath the point-estimate trajectory of Eq. (1).

## Discussion

The utilization of wastewater-based epidemiology surged during the COVID-19 pandemic with applications to outbreak detection and tracking temporal trends^8–10^. This report presents a major advance for wastewater surveillance by using a simple scaling model to directly estimate the unobservable fraction of persons in a population infected over time from SARS-CoV-2 RNA concentrations in wastewater. This approach circumvents problems with non-representative sampling inherent in observable COVID-19 cases, hospitalizations, or deaths, and in principle can be applied in any location where continuous wastewater sampling over time is possible.

There are some limitations to our study that invite further investigation. The data collection period in our research ended before the emergence of the Delta and Omicron coronavirus variants of concern. It is possible that the mean shedding time differs for such variants, which would imply a different time shift from the historical value assumed in our study, though at least one recent report has estimated that the mean generation times for the Alpha and Delta variants are similar to historical strains^11^. It is also possible that infection with different variants could change the scaling from SARS-CoV-2 concentrations to infections. Determining whether the mean shedding times and RNA concentrations differ for variants of concern relative to historical strains are topics for future research.

## Methods

The number of total tests and confirmed and probable COVID-19 cases was provided by the Connecticut Department of Public Health (CT DPH).

Nucleic acid was extracted from the primary sewage sludge of the New Haven, CT, USA wastewater treatment plant (which serves 200,000 residents), and SARS-CoV-2 RNA concentrations were quantified. Nucleic acid was extracted using commercial kits (Qiagen, RNeasy Powersoil Total RNA kit and Zymo, Quick-RNA Fecal/Soil Microbe Microprep). Nucleic acids were measured by spectrophotometry, the concentration adjusted to 200 ng µL^−1^ (NanoDrop, Thermo Fisher Scientific) and SARS-CoV-2 RNA concentrations were quantified through one-step qRT-PCR kit (BioRad iTaq™ Universal Probes One-Step Kit) using SARS-CoV-2 N1 and N2 primer sets for quantification in accordance with previously described protocols^6,12^. SARS-CoV-2 RNA concentrations were quantified daily throughout the study period. Further details regarding the construction of the SARS-CoV-2 RNA concentration dataset appear in the Supplementary Information.

There are two fundamental assumptions in our scaling model: 1. RNA concentrations provide a proportional measure of the extent of infection in the community given the population’s consistent discharge of fecal material into the local sewage collection system, and 2. the concentration of SARS-CoV-2 RNA in sewage sludge lags the population incidence of SARS-CoV-2 in accord with the generation time distribution (also referred to as the shedding load distribution) from infection to transmission, the mean of which is approximately 9 days^7,13^. Letting π_*t*_ denote the fraction of the population that is newly infected on day *t* (the incidence of infection) and ℓ denote the mean generation lag, the SARS-CoV-2 RNA concentration measured on day *t*, *Z*_*t*_, should approximately reflect the incidence of infection ℓ days earlier, that is, *Z*_*t*_ ≈ *k*π_*t*-ℓ_ where *k* is the constant of proportionality, and consequently the cumulative fraction of the population infected by the end of day t, given by ${C}_{t}=\sum_{j=0}^{t}{\pi }_{j}$, should approximately follow ${C}_{t}=k{^{\prime}}\sum_{j=0}^{t}{Z}_{j+\ell}$ where *k*′ = 1/*k* is the constant of proportionality scaling SARS-CoV-2 RNA concentration to infections per person. Given the cumulative number of infections *C*_t*_ as of some particular date t*, let ${S}_{t}=\sum_{j=0}^{t}{Z}_{t}$ denote the cumulative sludge RNA observed through day *t*. Then the scaling constant k′ can be evaluated from the relation ${C}_{{t}^{*}}=k{^{\prime}}{S}_{{t}^{*}+\ell}$ yielding ${k}^{^{\prime}}={C}_{{t}^{*}}/{S}_{{t}^{*}+\ell}$. Substituting back into the equation for cumulative incidence up to an arbitrary day *t* yields ${C}_{t}$ = ${(C}_{{t}^{*}}/{S}_{{t}^{*}+\ell})\times {S}_{t+\ell}.$ The cumulative incidence of infection in the 200,000 population served by the WWTP treatment plant was previously estimated as ${C}_{{t}^{*}}$ = 9.3% (95% CI [0.0643, 0.1217]) as of t^*^ = May 1, 2020 with a mean generation lag of 8.9 days^7^ which we round up to ℓ = 9. Substituting yields our scaling of cumulative RNA concentration to cumulative incidence shown in Fig. 2 as

1 $${C}_{t}=0.093\times {S}_{t+9}/{S}_{{t}^{*}+9}.$$

Confidence intervals follow from the variance of ${C}_{t}$ estimated via the delta method^14^.

## Supplementary Information

Supplementary Information 1.

Supplementary Information 2.

## Data Availability

All data employed in this report are available in the Excel file contained in the Source Data.
