# Supplementary material for: Scaling SARS-CoV-2 wastewater concentrations to population estimates of infection
Source: Sci Rep. 2022 Mar 3;12:3487. doi: 10.1038/s41598-022-07523-7 (PMC8894397; doi:10.1038/s41598-022-07523-7)
Supplement: Supplementary file 1 — Supplementary Information 1. [file 41598_2022_7523_MOESM1_ESM.pdf]

Supplementary Information

Scaling SARS-CoV-2 Wastewater Concentrations to Population Estimates of Infection

Edward H. Kaplan, Alessandro Zulli, Marcela Sanchez, Jordan Peccia

Extracting SARS-CoV-2 RNA from Wastewater and Scaling for Consistent RNA Measurement

From March 19, 2020 through September 30, 2020, well-mixed primary sludge was added directly to Qiagen's RNeasey PowerSoil Total RNA commercial extraction kit[1]. Supply problems required us to change the RNA extraction kit used to Zymo Quick-RNA Fecal/Soil Microbe Microprep for samples collected from October 1, 2020 to May 31, 2021[2]. To assure consistency between measurements based on these two different RNA extraction kits, Zymo extractions were also performed on stored samples originally analyzed with the Qiagen kit corresponding to 66 of the 74 days between March 19, 2020 and May 31, 2020 inclusive (eight of the original 74 samples were fully utilized and re-extraction with the Zymo kit was not possible). This enabled us to covert Qiagen RNA values to equivalent Zymo measurements as follows: letting Q_t and Z_t denote the SARS-CoV-2 RNA concentrations measured with the Qiagen and Zymo kits respectively on day t (t = 1, 2,…,66), we assumed that the Zymo RNA concentration on day t followed a normal distribution with mean $E(Z_t) = a + bQ_t$ and variance $Var(Z_t) = cE(Z_t)$ with the variance-proportional-to-mean formulation following from prior statistical analysis of SARS-CoV-2 RNA concentrations[3]. The parameters a, b and c were estimated by maximizing the log-likelihood function

$$\ln\mathcal{L} = -\frac{1}{2}\sum_{t=1}^{66}\left\{\ln(c(a+bQ_t)) + \frac{(Z_t-(a+bQ_t))^2}{c(a+bQ_t)}\right\}$$

using the Analytic Solver in Excel[4]. The covariance matrix of the estimated parameters was computed following standard maximum likelihood theory by inverting the negative Hessian matrix of the log likelihood function[5], and standard errors were extracted as the square root of the diagonal of the covariance matrix; these computations were also performed in Excel. The estimated intercept was not statistically different from zero ($\hat{a} = 9{,}180$, 95% CI [-6,870, 25,200]), so a model with no intercept (a = 0) was fit yielding $\hat{b} = 4.67$ (95% CI [4.04, 5.30]) and $\hat{c} = 30{,}300$ (95% CI [19,190, 41,400). The models with and without an intercept were further compared by computing twice the difference in the log likelihood values between the models yielding $\chi^2 = 2.2$ (p = 0.14 at 1 degree of freedom, not significant). Models with and without an intercept assuming that $Var(Z_t)$ is constant, corresponding to ordinary least squares regression analysis, were also estimated but these models produced substantially lower log likelihood values compared to the models described above (with the same number of estimated parameters) indicating substantially worse statistical fit, and were thus not considered further.

Assembling the Consistent Zymo RNA Concentration Dataset

We used the observed Zymo RNA concentrations together with the scaling model with no intercept described above to produce a consistent Zymo record of coronavirus RNA concentrations in the wastewater over the 439 days from March 19, 2020 to May 31, 2021. This was accomplished as follows:

1. On any day with an observed Zymo RNA concentration, that observed value was used. This accounts for 309 of the 439 days in the data.

2. On any day where a Qiagen measurement was made but no corresponding Zymo measurement was available, a Zymo value was estimated as of 4.67 times the associated Qiagen value based on the scaling model described above. This accounts for 125 of the 439 days in the data.

3. On any day missing both Qiagen and Zymo values (corresponding to the five days during the study period that the treatment plant did not provide a sample out of 439 days total), missing Qiagen values were interpolated from nearby observed values, and these interpolated values were then multiplied by 4.67 to produce an estimated Zymo value in accord with the scaling model described above. Specifically: Qiagen values missing on May 3 and May 6 of 2020 were interpolated by averaging the observed Qiagen values two days before and two days after the missing values, while Qiagen values missing on July 20, 21 and 22 of 2020 were interpolated linearly as 0.25, 0.5, and 0.75 times the distance respectively from the observed Qiagen value on July 19 to the observed value on July 23.

The consistent Zymo dataset appears in Dataset S1, and indicates which values were measured directly or scaled from Qiagen measurements (and for the latter which Qiagen values were observed versus interpolated and the means of interpolation).

Source Data (separate file). Date, Number of COVID-19 Tests, Number of Positive COVID-19 Tests, Qiagen RNA Concentration, Zymo RNA Concentration, Cumulative Zymo RNA Concentration, Wastewater SARS-CoV-2 Cumulative Incidence (Equation (1)), Upper 95% CI, Lower 95% CI, Estimated SARS-CoV-2 Cumulative Incidence from Models 1, 2 and 3

References:

1. Peccia, J. et al. Measurement of SARS-CoV-2 RNA in wastewater tracks community infection dynamics. *Nature Biotechnology* **38**, 1164–1167 (2020). https://www.nature.com/articles/s41587-020-0684-z (accessed 14 July 2021).
2. Zulli, A. et al. Predicting daily COVID-19 case rates from SARS-CoV-2 RNA concentrations across a diversity of wastewater catchments. In press with *FEMS Microbes*, preprint at *medRxiv* https://www.medrxiv.org/content/10.1101/2021.04.27.21256140v1 (2021, accessed 14 July 2021).
3. Kaplan, E. H. et al. Aligning SARS-CoV-2 indicators via an epidemic model: application to hospital admissions and RNA detection in sewage sludge. *Health Care Management Science* (2020). https://link.springer.com/article/10.1007/s10729-020-09525-1 (accessed 14 July 2021).
4. Frontline Systems, Analytic Solver® for Excel. https://www.solver.com/analytic-solver-platform (accessed 14 July 2021).
5. Cox, D. R. & Hinkley, D. V. *Theoretical Statistics* (Chapman and Hall, 1974).
